# Notes of a Case of Latent Phthisis Pulmonalis Terminating Suddenly in Pneumothorax

**Published:** 1852-05

**Authors:** F. W. Sargent

**Affiliations:** The Physicians to the Girard College


### MEDICAL EXAMINER,

AND

### RECORD OF MEDICAL SCIENCE.

NEW SERIES.—NO. LXXXIX.—MAY, 1852.

---

### ORIGINAL COMMUNICATIONS.

---

*Notes of a Case of latent Phthisis pulmonalis terminating suddenly in Pneumothorax.* By F. W. Sargent, M. D., one of the Physicians to the Girard College.

Wm. McLaughlin, æt. 13, lost his parents and several brothers and sisters from consumption. He was rather under the ordinary size of boys of his age, possessed of but little muscular vigor, and was indisposed to indulge in the out-of-door sports of his companions. During the three years of his residence at the Girard College, he was not at any time sick, though he was always looked upon as "delicate." He had had a slight cough from time to time, after special exposure ; but he had no chronic or habitual cough, as I am assured by the Governess who had charge of the division in which he was, and who, at night, occupied a room adjoining that in which he slept. And no one of his friends recollected his having been severely sick at any time previous to his admission to the College. His skin was thin and white ; his face freckled ; his hair of a bright red color ; mental powers not much above mediocrity.

The first occasion of his being in the Infirmary for medical treatment, was on Saturday, December 20th, 1851, when he complained simply of feeling a little unwell, without giving any definiteness to his complaints; this was in the evening, and he had in the morning voluntarily accompanied some visitors to the top of the college building, a feat which involves considerable fatigue to those who are feeble. He remarked that he thought he had taken cold from exposure on the roof of the College, where there is always a considerable breeze.

He slept soundly on Saturday night, and on Sunday morning said he felt quite well. He informed the nurse that he had not had an evacuation of his bowels on the previous day, and she accordingly gave him a dose of castor oil, which he immediately rejected from his stomach; and as he continued to feel sick, with a sensation of oppression at the epigastrium, a teaspoonful of Coxe's hive syrup was administered, which he threw up speedily, and with relief to these symptoms. The act of vomiting in both instances was easy and unattended with straining.

Toward evening, he became feverish, and I was sent for to see him. He complained of soreness and pain at the lower part of his chest, in front and on the left side, aggravated by coughing or any violent or prolonged action of the respiratory muscles, but not partaking of the characters of the pain of decided pleurisy. His cough was frequent, hard and dry; pulse frequent; skin hot and moist. His decubitus was easy, though he preferred lying on his back or on the right side. The percussion was resonant, and about equally so on both sides of his chest; the respiratory murmur was somewhat more feeble and less completely developed on the left than on the right, unmixed with râle of any kind, however. In the examination of his chest I was struck with his thinness; there seemed to be no fat about his chest or arms, and the muscles were small and soft.

In the absence of any positive indication of inflammation of the lungs, bronchial tubes, or pleuræ, and hoping that the case would prove to be a rheumatic attack affecting chiefly the parietes of the thorax, I directed that his chest should be well bathed with a warm liniment composed of laudanum, spirits of camphor, and olive oil, and subsequently covered with a warm poultice of hops—both to be repeated from time to time; and that he should take, every two or three hours, according to circumstances, 1 gr. of nitrate of potassa, with 2 grs. of Dover's powder.

He slept none during the night, but coughed very frequently and with violence, without expectoration ; the soreness and pain in his chest continued but little abated by the applications which were made, and by the Dover's powder; although there was no great aggravation of these symptoms, nor any sudden accession. Early in the morning, however, he complained of increased soreness at the top of his left chest, and his cough became rather more violent in its paroxysms.

On my visit at 9 A. M., Monday morning, I found his breathing more frequent and more labored ; his cough also increased; the pulse more frequent, and the skin hotter ; the left side of the chest was tympanitic on percussion, and distended with air which elevated the intercostal depressions, and rendered this side of the thorax more full than the other ; the heart was a little displaced towards the median line of the sternum, and the vesicular murmur was replaced by bronchial respiration, amphoric respiration, and, occasionally, "metallic tinkling." In short there was undoubted "*pneumothorax.*" And, in view of the boy's appearance and history, and the fate of his parents and other relatives, there could be little question that a *tubercular cavity* had opened into the pleural sac.

A blister was applied to the left side of the chest; the nitre and Dover's powder were continued, arrow-root boiled with milk and chicken-tea were directed to be given in small portions and as frequently as he might desire; (he had no appetite, and would take but little nourishment.)

In the evening his condition seemed to be about the same as in the morning. Conditional directions were given as to the administration of nourishment, and, in addition, of wine-whey.

During the night he slept at intervals, on one occasion as long as an hour and a half, being awakened by cough and dyspnœa. But at 6½ A. M., Tuesday, he suddenly experienced a sensation of increased oppression, and immediately said he thought he was dying. He died in the course of an hour from this time, his intellect clear to within a few minutes of his death.

*Examination twenty-six hours after death.*

Great emaciation of the muscles and integuments of the trunk, and limbs ; posterior surface stained.

Both sides of chest resonant, left side tympanitic, and measuring in circumference three fourths of an inch at its fullest part more than the right. A small puncture was made through the parietes of the left chest, and air rushed out audibly, and with sufficient force and volume to extinguish a lighted candle held near the orifice, the walls of the thorax on this side proportionally subsiding.

The left pleural cavity contained 1½ to 2 ounces of limpid yellowish fluid; soft lymph was adherent to the pulmonary pleura, and in less amount to the costal also, in flakes and more diffused patches, and the membrane was reddened in spots. The lung had collapsed, containing little or no air; but it was perfectly free from any adhesion, excepting at a circumscribed spot just below the apex, in front, where there was a firm, fleshy looking mass of fibrin, about a quarter of an inch in length, which connected the two surfaces of the pleura. Immediately beneath this adhesion was the perforation. The orifice was about as large as a crow's quill, round, its margins thin; it communicated, through the pleura, which was two lines in thickness, with an irregularly-shaped cavity as large as a hazel nut, free from any adventitious lining membrane, containing no secretion, and opening into a very small bronchial twig. The pulmonary tissue around this cavity was in an apparently healthy condition, excepting that it was of a dusky red color. At different points in the lung were several small crude, unsoftened tubercles; no tubercular granulations.

The right pulmonary pleura was closely adherent to the walls of the chest, by firm, old adventitious tissue, without any evidence of recent inflammation. The lung was filled with air, and its texture generally presented no abnormal appearance, excepting from a moderate degree of vascular turgescence, and the presence of a few crude, unsoftened tubercles scattered here and there, in the midst of a crepitating pulmonary tissue. Near the apex, posteriorly, was, moreover, a small cavity of rather greater capacity than that in the left lung; around it, the texture was normally pliable and aerated; the outer wall of the vomica consisted merely of the pleura, which was thickened; the adhesions upon this side probably saved the boy from *pneumothorax* of the right cavity.

The lining membrane of the bronchial tube was uninflamed, although somewhat injected here and there.

The bronchial glands were generally enlarged, some of them tuberculous. No tubercles were found in the other organs.

The space already taken up by the simple narration of this case, precludes anything more than a mere allusion to its most interesting features. These are, the insidious manner in which the tuberculosis had progressed; the absence of symptoms, so far as we can learn, of the pleurisy of the right side, or of the local pleurisy at the summit of the left chest; the protective influence of the right pleurisy, as respects the superficial cavity in the corresponding lung; the occurrence of the perforation of the left pulmonary pleura, and the rapid supervention of the fatal termination. A *very minute* physical examination of the apices of the lungs, during life, would probably have revealed the existence of the cavities there; but this knowledge would scarcely have availed anything with reference to the treatment of the disease, or to the prevention of the pneumothorax. From all the facts of the case, it seems most reasonable to suppose that the rupture of the left pleura over the abscess, occurred during an act of violent coughing, early on Monday morning.

